# Hydrogen sulfide inhibits aortic valve calcification in heart via regulating RUNX2 by NF-κB, a link between inflammation and mineralization

**DOI:** 10.1016/j.jare.2020.07.005

**Published:** 2020-07-21

**Authors:** Katalin Éva Sikura, Zsolt Combi, László Potor, Tamás Szerafin, Zoltán Hendrik, Gábor Méhes, Péter Gergely, Matthew Whiteman, Lívia Beke, Ibolya Fürtös, György Balla, József Balla

**Affiliations:** a Division of Nephrology, Department of Medicine, Faculty of Medicine, University of Debrecen, 4012 Debrecen, Hungary; b HAS-UD Vascular Biology and Myocardial Pathophysiology Research Group, Hungarian Academy of Sciences, Debrecen, Hungary; c Department of Cardiac Surgery, Faculty of Medicine, University of Debrecen, 4012 Debrecen, Hungary; d Department of Pathology, University of Debrecen, Faculty of Medicine, 4012 Debrecen, Hungary; e Department of Forensic Medicine, Faculty of Medicine, University of Debrecen, Debrecen, Hungary; f University of Exeter Medical School, St. Luke’s Campus, Magdalen Road, Exeter EX1 2LU, UK; g Department of Pediatrics, Faculty of Medicine, University of Debrecen, 4012 Debrecen, Hungary

**Keywords:** H_2_S, AP72, Aortic valve, Inflammation, CAVD, Apolipoprotein E knockout mice, AS, stenotic aortic valve with calcification, HAV, healthy aortic valve from suicide patients, cVIC, control healthy valve interstitial cells, VIC, valvular interstitial cells, mHAV, healthy mouse aortic valve, mVIC, mouse valvular interstitial cells, AP72, 4-methoxyphenyl piperidinylphosphinodithioc acid, ApoE^-/-^, apolipoprotein E-deficient mice, STED, Stimulated Emission Depletion Nanoscopy, CSE, Cystathionine gamma-lyase, CBS, Cystathionine beta-synthase, CAVD, calcific aortic valve disease, IL-1β, interleukin-1β, TNF-α, tumor necrosis factor α, NF-κB, nuclear factor-κB

## Abstract

**Introduction:**

Hydrogen sulfide (H_2_S) was revealed to inhibit aortic valve calcification and inflammation was implicated in the pathogenesis of calcific aortic valve disease (CAVD).

**Objectives:**

We investigate whether H_2_S inhibits mineralization via abolishing inflammation.

**Methods and results:**

Expression of pro-inflammatory cytokines, interleukin-1β (IL-1β) and tumor necrosis factor α (TNF-α) were increased in patients with CAVD and in calcified aortic valve of ApoE-/- mice. Administration of H_2__2_S releasing donor (4-methoxyphenyl piperidinylphosphinodithioc acid (AP72)) exhibited inhibition on both calcification and inflammation in aortic valve of apolipoprotein E knockout mice (ApoE-/-) mice is reflected by lowering IL-1β and TNF-α levels. Accordingly, AP72 prevented the accumulation of extracellular calcium deposition and decreased nuclear translocation of nuclear factor-κB (NF-κB) in human valvular interstitial cells (VIC). This was also accompanied by reduced cytokine response. Double-silencing of endogenous H_2_S producing enzymes, Cystathionine gamma-lyase (CSE) and Cystathionine beta-synthase (CBS) in VIC exerted enhanced mineralization and higher levels of IL-1β and TNF-α. Importantly, silencing NF-κB gene or its pharmacological inhibition prevented nuclear translocation of runt-related transcription factor 2 (Runx2) and subsequently the calcification of human VIC. Increased levels of NF-κB and Runx2 and their nuclear accumulation occurred in ApoE-/- mice with a high-fat diet. Administration of AP72 decreased the expression of NF-κB and prevented its nuclear translocation in VIC of ApoE-/- mice on a high-fat diet, and that was accompanied by a lowered pro-inflammatory cytokine level. Similarly, activation of Runx2 did not occur in VIC of ApoE-/- mice treated with H_2_S donor. Employing Stimulated Emission Depletion (STED) nanoscopy, a strong colocalization of NF-κB and Runx2 was detected during the progression of valvular calcification.

**Conclusions:**

Hydrogen sulfide inhibits inflammation and calcification of aortic valve. Our study suggests that the regulation of Runx2 by hydrogen sulfide (CSE/CBS) occurs via NF-κB establishing a link between inflammation and mineralization in vascular calcification.

## Highlights

• H_2_S derived from CSE/CBS inhibits inflammation and calcification of valvular interstitial cells.
• H_2_S releasing molecules inhibits inflammation and calcification of valvular interstitial cells.
• H_2_S inhibits inflammation and calcification in aortic valve of apolipoprotein E deficient mice.
• H_2_S suppresses nuclear translocation of NF-κB and subsequent expression of IL-1β and TNF-α.
• Activation of Runx2, its nuclear translocation is mediated by NF-κB in calcifying conditions.
• Inflammation and calcification are connected via master transcription factors, NF-κB and Runx2.

## Graphical abstract

**Figure:**
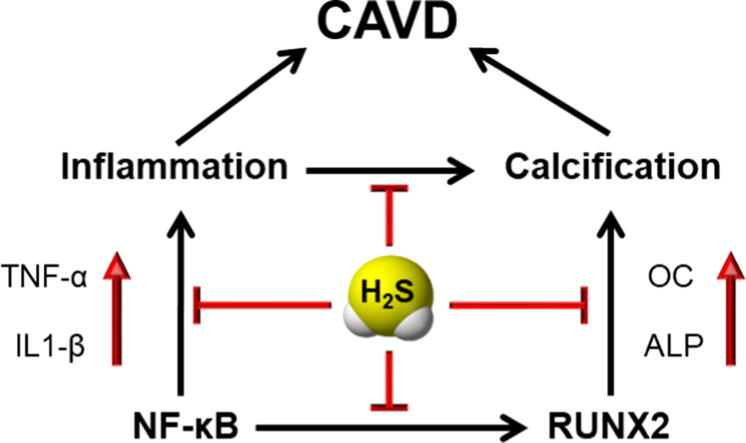

## Introduction

Calcific aortic valve disease (CAVD) is the most common indication for surgical aortic valve replacement in the world [1]. CAVD is particularly dominant in national populations over age 65 (2–7%) [2] and also a frequent disorder in chronic kidney disease (CKD) [3]. The percentage of CAVD has been increasing in the last 10 years and the incidence of the disease expected to increase in the future [4], [5], [6], [7]. Surgical intervention remains the only available option to treat CAVD while efficient conservative therapy is lacking [8], [9], [10], [11], [12], [13].

In calcific aortic valve disease, the valvular interstitial cells transdifferentiate into osteoblast‐like cells contributing to the mineralization of tissue [1], [9]. Nuclear translocation of osteogenic transcription factor Runx2 initiates the transition of cells towards an osteoblast phenotype in response to various pathological stimuli such as high phosphate [14].

It has been revealed by O’Brien et al. that pro-inflammatory cytokines, interleukin-1β (IL-1β) and tumor necrosis factor α (TNF-α) are associated with CAVD, and inflammation is a hallmark of CAVD [15], [16], [17]. Furthermore, Rajamannan and colleagues in the National Heart, Lung, and Blood Institute provided novel insights of CAVD. They demonstrated that CAVD is an active complex osteogenic process and inflammation plays a central role in its initiation and progression. It was shown that abnormal hemodynamic forces such as shear stress, hypertension or elevated stretch experienced by the valve leaflets can cause tissue remodeling and inflammation, consequently may lead to calcification, stenosis and heart valve failure [1], [2], [9]. Additionally, immunohistochemistry analysis, based on 285 CAVD patient’s heart valve, indicated that 28.4% of affected specimens of CAVD are characterized by massive leukocytes infiltration and high expression of TNF-α as a resulting chronic inflammation [17]. In an animal model of IL-1Ra (interleukin-1 receptor antagonist) deficiency in mice an enflamed aortic valve stenosis was shown to occur, and TNF-α participated in the pathogenesis of valvular mineralization [18].

The induction of the signaling pathway of nuclear factor-κB (NF-κB) is one of the main mediators of inflammation that plays a critical role in the development and progression of CAVD [19]. Nuclear translocation of NF-κB induced by TNF-α promotes inorganic phosphate-induced calcification in human aortic smooth muscle cells [20].

H_2_S is a third gasotransmitter among nitrogen-oxide and carbon-monoxide and has various potential functions in physiological and pathological conditions including CAVD [21]. In our previous studies we revealed that H_2_S inhibits osteoblastic transdifferentiation of vascular smooth muscle cells and valvular interstitial cells and abrogates calcification of aortic heart valve in ApoE deficiency in mice. Furthermore, H_2_S generation was found to be reduced in valvular tissue in CAVD. We identified three separate anti-calcification pathways for H_2_S: i, inhibits inorganic phosphate uptake, ii, prevents nuclear translocation of Runx2 and iii, increase pyrophosphate level.

The aim of this study was to examine whether inhibition of aortic valve calcification by H_2_S occurs via exerting anti-inflammatory action.

## Material and methods

### Materials

All chemicals were analytical reagent grade or higher and obtained from Sigma-Aldrich, (St Louis, MO, USA). The sulfide donor molecule used in this work − AP72 (4 methoxyphenyl) (piperidin-1-yl) phosphinodithioc acid) – was synthesized in-house [22], [23], [24]. Sulfide stock solutions were prepared fresh daily in water.

### Cell isolation and culture

Cusps of human aortic valve leaflets were obtained between December 2018 to December 2019 (48 patients) (Regional Research Ethical Committee, Project No.: 3853–2013 and 4699–2016) from surgical specimens from patients undergoing complete aortic valve replacement for significant stenosis with calcification (referred to as aortic valve with stenosis (AS) for this study). Cells were isolated from human heart valves by collagenase (600 U/mL) (EMD Millipore Corp.; 234155-100MG) and cultured as previously described [25]. Since phenols are capable to absorb a high amount of H_2_S from liquids [26] our experiments were carried out in phenol red-free media.

### Healthy heart valves

Healthy aortic valves (HAV) for controls were obtained from cadavers (N = 3) of suicide or traumatic events without cardiovascular diseases from Department of Forensic Medicine, University of Debrecen (Regional Research Ethical Committee, Project No.: 5038–2018).

### Control VIC primary cells

cVIC cells were purchased from Innoprot Ltd. (Parque Tecnológico de Bizkaia, Batch #0960; Spain). The cells were derived from a healthy 8 years old Caucasian male.

### Animals

All procedures followed were in accordance with the ethical standards of the responsible committee on human experimentation (institutional and national) and with the Helsinki Declaration of 1975, as revised in 2008. Informed consent was obtained from all patients for being included in the study. Animal experiments performed in this study were approved by the Scientific and Research Ethics Committee of the Scientific Council of Health of the Hungarian Government under the registration number of DE MÁB/157–5/2010 and the Institutional and National Guidelines for the care and use of animals (fisheries) were followed. C57BL/6 ApoE^-/-^ mice were maintained at the University of Debrecen under specific pathogen-free conditions in accordance with guidelines from the Institutional Ethical Committee. Mice were randomly divided into four groups. Non-high fat diet group (N = 7) received a standard chow diet. To induce aortic valve inflammation and/or calcification, mice were kept atherogenic diet (15% fat, 1.25% cholesterol, ssniffSpezialdiäten GmbH, Soest, Germany) at the age of 8 weeks. Parallel with the atherogenic diet mouse was injected intraperitoneally with AP72 (266 µmol/kg body weight; N = 7) or vehicle (saline; N = 7) in every other day as previously described. Aortas were collected after 8 weeks of treatment. For the detection of the inflammatory process in vivo, mice were euthanized every second week until 8 weeks. All mice were euthanized by a predictable and controllable administering slow-fill compressed CO_2_ asphyxiation. Atherogenic food composition (high-fat diet): Crude Nutrients (%): Crude protein 19%; crude fat 15.2%; crude fiber 3.4%; crude ash 6.3%; starch 25.6%; sugar 11.2%; Additives (per kg): vitamin A 15,000 IU; vitamin D3 1,000 IU; vitamin E 110 mg; vitamin K3 5 mg; vitamin C 0 mg; copper 13 mg.

### Alizarin Red s staining for determination of extracellular calcium deposition

Alizarin Red S (Sigma Aldrich; A5533) staining was used to visualize the calcium deposition. Plates were fixed with 3% paraformaldehyde and stored at 4 °C for 10 min and stained with a 2% solution of Alizarin Red S. All calcific nodules in each well were then manually counted under a microscope. To quantify the calcified area we used imageJ software.

### Western blot analysis

To detect NF-κB, Runx2, TNF-α and IL1-β were used rabbit anti-human TNF-α (Thermo Fisher Scientific; PA5-19810; 400 ng/mL); rabbit anti-human IL1-β (Invitrogen; 17h18l16; 400 ng/mL), rabbit anti-human Runx2 (Proteintech; 20700–1-AP; 60 ng/mL), and rabbit anti-human NF-κB (Cell Signaling Technology; D14E12; 400 ng/mL). Next, HRP-labeled anti-rabbit IgG antibody was used as a secondary antibody. Complexes of antigen–antibody were visualized with a horseradish peroxidase chemiluminescence detection system (Amersham Biosciences; RPN2109). Membranes were reprobedwith glyceraldehyde-3-phosphate dehydrogenase (GAPDH).

### Quantitative Real-Time PCR (qRT-PCR)

VIC were cultured in growth media or calcification media supplemented with 20 µmol/L AP72. After 5 days cells were harvested. Total RNA was isolated using RNAzol STAT-60 according to the manufacturer’s instructions (TEL-TEST Inc., Friendswood, TX, USA). RNA concentration was measured with NanoDropTM 2000c spectrophotometer (Thermo Scientific Inc., Waltham, MA, USA). Subsequently, cDNA synthesis was performed using a high-capacity cDNA kit (Applied Biosystems, Foster City, CA). We used real-time PCR technique for quantification of mRNA levels of IL1-β and TNF-α (Thermo Fisher Scientific Inc.) and GAPDH (Thermo Fisher Scientific Inc.). TaqMan Universal PCR Master Mix was purchased from Applied Biosystems (Applied Biosystems, Foster City, CA). Finally, we performed TaqMan quantitative PCR (40 cycles at 95° C for 15 sec. and 60° C for 1 min.) on 96-well plates with the Bio-Rad CFX96 (Bio-Rad Laboratories Inc., Hercules, California, USA) detection system. Results were expressed as mRNA expression normalized to GAPDH.

### Immunohistochemistry

Heart valve tissues were fixed with formaldehyde for one day followed by TRIS buffer and embedded in paraffin wax. Subsequently, slides were deparaffinized in xylene for 5 min and then rehydrated. For immunohistochemistry, slides were subjected to a peroxidase-blocking reagent for 5 min (3% hydrogen peroxide was used to block endogenous peroxidase activity). Antigen retrieval was performed in an epitope retrieval solution (Leica RE-7113) at pH 6 using a pressure cooker (rice programs, IDA Avair 6 L pressure cooker). Von Kossa staining and the follow IHC stains were performed with the following antibodies: rabbit anti-human TNF-α (Thermo Fisher Scientific; PA5-19810; 400 ng/mL); rabbit anti-human IL1-β (Invitrogen; 17h18l16; 400 ng/mL). Antibody binding was visualized by the Super Sensitive TM One Step Polymer-HRP IHC Detection System. The intensity and distribution of antibodies expression were assessed by light microscopy (Leica DM2500 microscope, DFC 420 camera and Leica Application Suite V3 software, Wetzlar, Germany).

### Immunohistochemistry from mouse heart valves

Briefly, mouse heart valve tissues were fixed in formaldehyde for one day followed by TRIS buffer and embedded in paraffin wax. Subsequently, slides were deparaffinized in xylene and then rehydrated. For immunohistochemistry, slides were subjected to the peroxidase-blocking reagent. Von Kossa staining and the following stains were performed with the following primary antibodies: anti-IL-1β antibody (Invitrogen; 17h18l16; 400 ng/mL), anti-TNF-α antibody (Thermo Fisher Scientific; PA5-19810; (400 ng/mL). Antibody binding was visualized by the Super Sensitive TM One Step Polymer-HRP IHC Detection System. Liquid DAB chromogen (BG-QD630-XAKm BioGenex) were added for samples. The intensity and distribution of antibodies expression were assessed by light microscopy (Leica DM2500 microscope, DFC 420 camera and Leica Application Suite V3 software, Wetzlar, Germany).

### Immunofluorescence staining

VIC were cultured on the coverslip and treated with or without calcification medium in the absence of phenol red, supplemented with 20 µmol/L AP72 for 5 days. After treatment, the cells were fixed with 3.7% formaldehyde for 15 min. After fixation, cells were blocked with 10% goat serum for 1 h at room temperature. Rabbit polyclonal anti-human NF-κB (Cell Signaling Technology; D14E12; 400 ng/mL) was used as a primary antibody to showing NF-κB localization in VIC. Primary antibodies labeled with goat anti-rabbit Alexa 488 (Thermo Fisher Scientific, A11070) as fluorophore for 1 h in dark at room temperature. Hoechst was used to stain nuclei. Multicolor STED imaging was acquired with STED (Stimulated Emission Depletion) Leica TCS SP8 gated STED-CW nanoscopy (Leica Microsystem Mannheim, Germany). Gated STED images were deconvolved using Huygens Professional (Scientific Volume Imaging B.V., Hilversum, Netherlands) software. Colocalization rate of NF-κB of the samples were measured by Image J software.

### Nuclear and cytoplasmic protein extraction

Cells were cultured in growth medium and treated with or without calcification medium supplemented with 20 μmol/L AP72. After treatment, cells were harvested with cell scraper and collected into a centrifuge tube. Pellets were washed twice with PBS and then ice‐cold 1 × cytoplasmic lysis buffer (20 mmol/L Tris–HCl pH 8.0, 100 mmol/L NaCl, 300 mmol/L sucrose, 3 mmol/L MgCl_2_, protease inhibitor cocktail) was added to the pellets. Cell suspensions were incubated on ice for 15 min. After centrifugation, the supernatants were collected (contains cytoplasmic proteins); the pellets were washed with PBS and resuspended in ice‐cold nuclear extraction buffer (20 mmol/L Tris–HCl pH 8.0, 300 mmol/L NaCl, 2 mmol/L EDTA pH 8.0, protease inhibitor cocktail). After that, the samples were passed five times through a 27 gauge needle, for the extraction of the nuclear proteins followed by centrifugation at 8,000 × g, 4 °C for 20 min. The supernatant contains the nuclear fraction. The protein concentration of the samples was determined by the BCA Protein Detection Kit (Amersham).

### CSE, CBS and NF-κB gene silencing

CSE, CBS and NF-κB genes were silenced, using appropriate siRNAs. Briefly, the VIC were cultured on 12‐well plates in antibiotic‐free medium (DMEM, Sigma). At about 70% of confluence, cells were transfected with siRNA against CSE and CBS (CSE: Ambion, 4392420; s3710; CBS: Ambion, 4390824; s289). NF-κB gene silencing using siRNA (Ambion, 4392420; s9505) was performed. Transfection occurred for 4 hr in minimal serum‐content medium (Opti‐MEM; Gibco). At the end of transfection, 30% FBS containing antibiotic‐free DMEM was added. Next day, cells were washed and treated with AP72 every second day until 5 days.

### Pharmacological inhibition of NF-κB

VIC were cultured in 12‐well plates in growth medium or calcification medium in the absence of phenol-red. Inhibition of NF-κB was carried out with the relevant inhibitor SC75741 (Sigma Aldrich, Cat# SML2382-MG). The inhibitor was used at 5 nmol/L).

### Statistical analysis

Data were analyzed by GraphPad Prism 5.02 software (GraphPad Software Inc., 7825 Fay Avenue, Suite 230 La Jolla, CA 92037). All statistical data are expressed as mean ± SEM. If data groups passed normality test and equal variance test, we performed Student's *t*-test or One Way ANOVA followed by Bonferroni post hoc tests as indicated in figure legends. P < 0.05 was considered significant.

## Results

### Pro-inflammatory cytokines are expressed in human calcified stenotic aortic valves

**Fig. 1:**
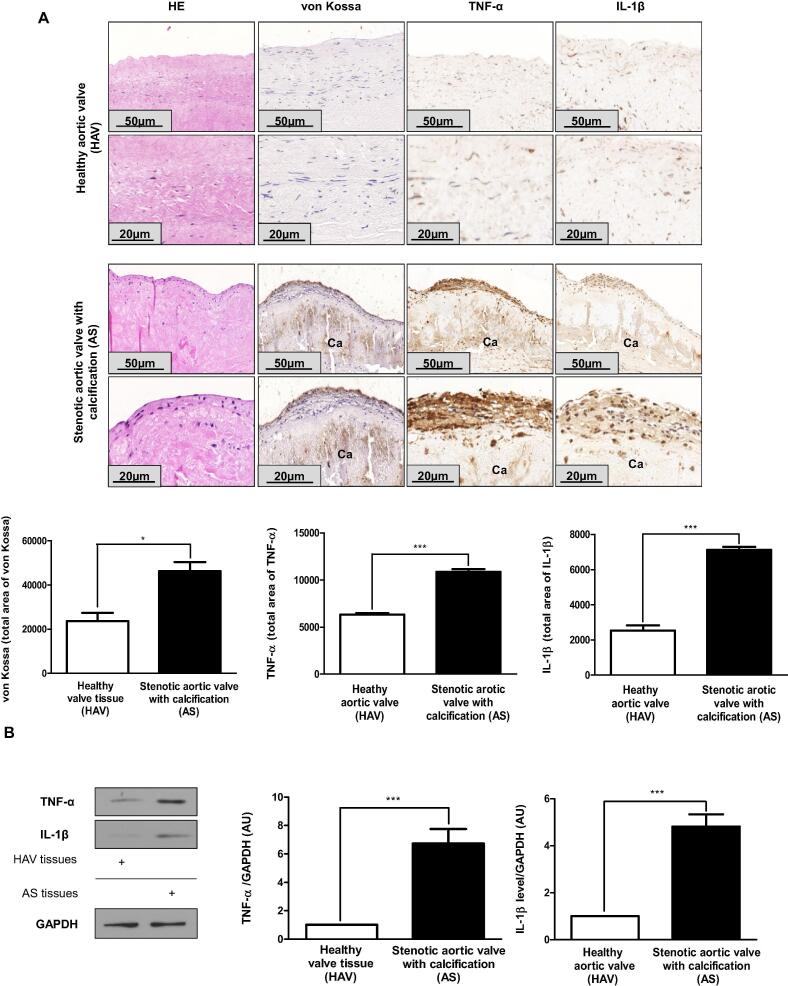
Inflammatory cytokines are characteristic of human stenotic aortic valve. A) Hematoxylin and eosin, von Kossa, TNF-α and IL1-β IHC staining were performed on HAV valves derived from the Department of Forensic Institute, University of Debrecen (upper images; N = 3) and on AS valves (lower images, N = 5). Scale bars (50 µm and 20 µm) and pixel intensity of IHC staining were shown. Representative staining was shown. B) IL1-β and TNF-α protein expression in HAV and AS heart valve tissue was assessed by western blot analysis. Protein expressions were normalized to GAPDH. Results were analyzed by One Way ANOVA, Bonferroni’s Multiple Comparison Test and were shown as mean values ± SEM of five independent experiments. *P < 0.01; ***P < 0.0001.

To show inflammation is present in human calcified stenotic aortic valves (AS), we stained heart valves derived from patient underwent aortic valve replacement for cytokines, IL-1β and TNF-α. For controls, we obtained healthy aortic valves (HAV) from cadavers of suicide or traumatic events without cardiovascular diseases. Strong von Kossa staining indicated a severe valvular calcification in AS. As shown in Fig. 1A, mineralization was accompanied by increased expression of IL-1β and TNF-α in AS compared to HAV. Western blot analysis also demonstrated higher levels of pro-inflammatory cytokines (IL1-β and TNF-α) in AS compared to HAV (Fig. 1B). These data are accordance with the previously revealed observations, namely inflammation and calcification are hallmarks in CAVD [1].

### Exogenous H_2_S prevents inflammation in aortic valves of apolipoprotein E deficient mice

**Fig. 2:**
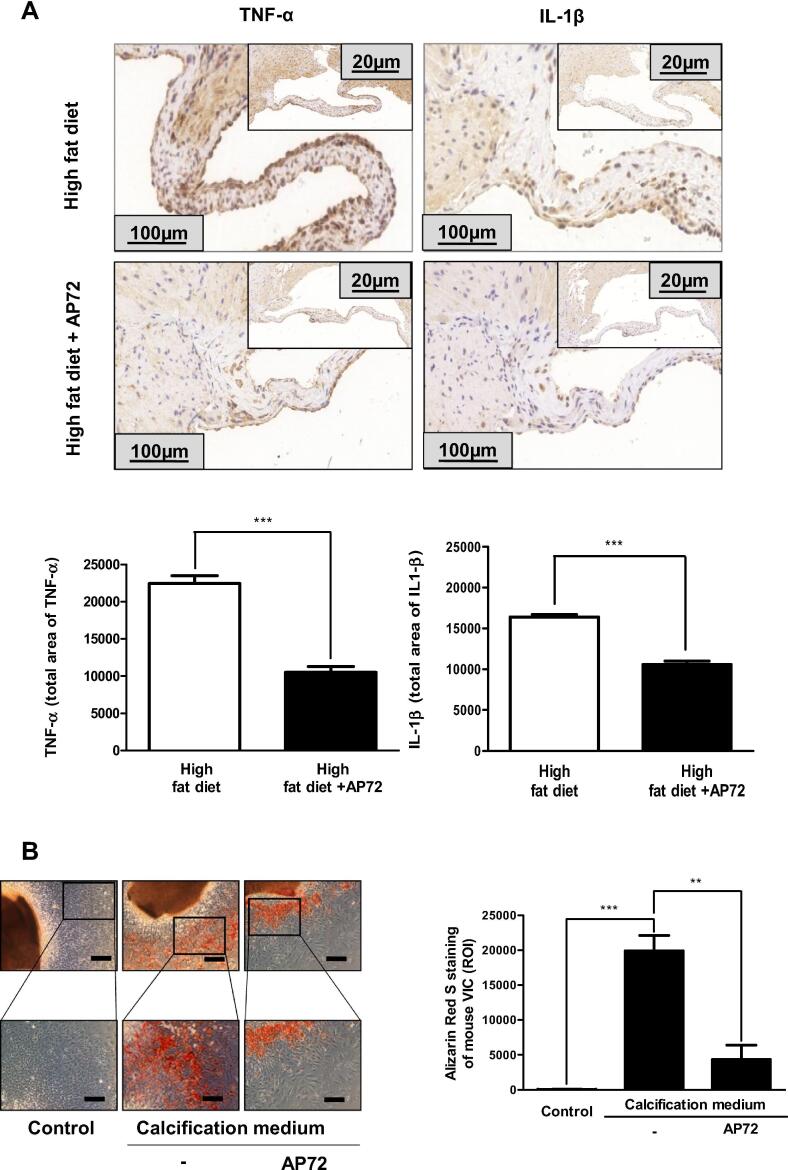
Hydrogen sulfide inhibits inflammation and calcification. A) TNF-α and IL1-β staining were performed on aortic valves of ApoE^-/-^ mice kept on high-fat diet (upper panels, N = 5) and on high-fat diet treated with AP72 (lower panels; N = 5). B) Alizarin Red S staining was performed in ex vivo cultured aortic valves of ApoE^-/-^ mice cultured in growth medium or calcification medium in the absence or presence with AP72 were shown. Results were analyzed by One Way ANOVA, Bonferroni’s Multiple Comparison Test and were shown as mean values ± SEM of five independent experiments. **P < 0.001; ***P < 0.0001.

Since we previously demonstrated that H_2_S inhibits aortic valve calcification in apolipoprotein E deficient mice (ApoE^-/-^ mice) kept on a high-fat diet [3], [27] we tested whether it also affects inflammation of aortic valves of ApoE^-/-^ mice kept on a high-fat diet. We utilized AP72, a slow-releasing H_2_S donor, previously found to be a potent inhibitor of valvular mineralization, in our experiments [3]. Administration of AP72 intraperitoneally significantly inhibited the thickening of the aortic valves and the expression of inflammatory cytokines IL1-β and TNF-α (Fig. 2A). Similarly, to human AS (Fig. 1A), the number of cells stained for IL1-β and TNF-α were pronounced in aortic valves of ApoE^-/-^ mice fed with a high-fat diet. As shown in Fig. 2A, AP72 treatment significantly reduced the expression of both IL1-β and TNF-α in aortic valve tissue of ApoE^-/-^ mice fed with a high-fat diet. Similar findings were observed in *ex vivo* conditions, as we employed high phosphate exposure to provoke calcification in valves of ApoE^-/-^ mice. Alizarin Red S staining demonstrated a significant attenuation of calcification of valvular interstitial cells (Fig. 2B) treated with AP72.

**Fig. 3:**
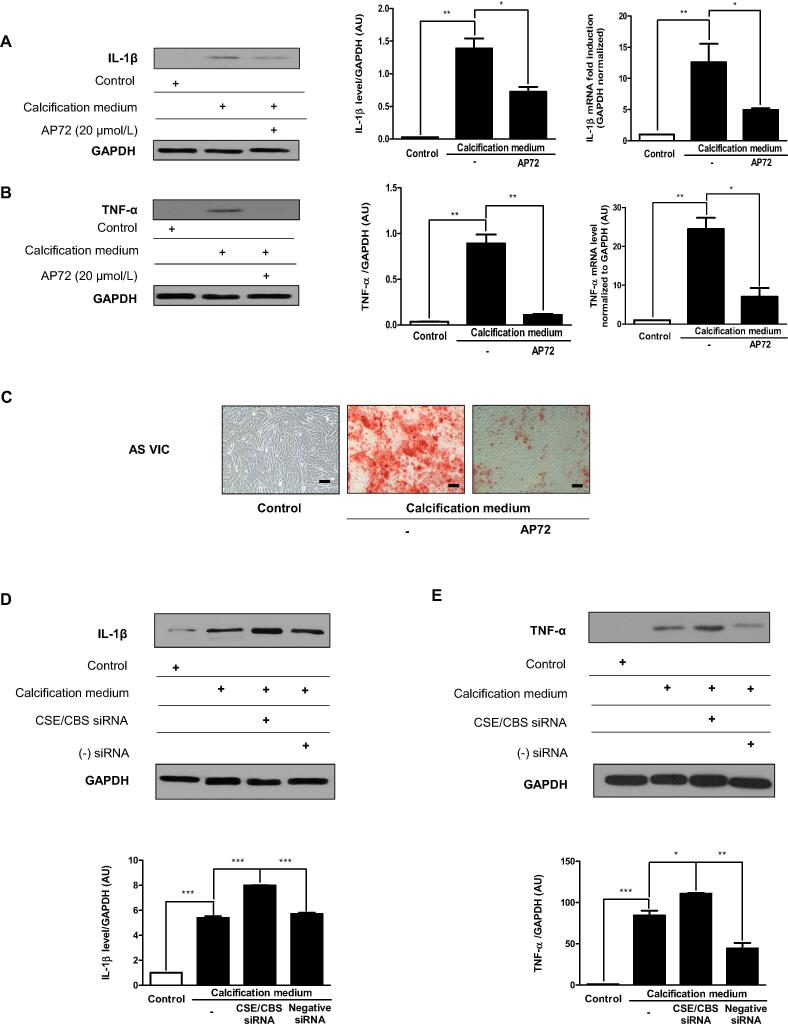
Hydrogen sulfide controls pro-inflammatory cytokines expression. A) IL1-β, B) TNF-α western blot and RT-qPCR were performed and normalized to GAPDH. C) VIC cultured in growth medium or calcification medium in the absence or presence with AP72. Representative Alizarin Red S staining of human AS derived VIC was shown. VIC were cultured in growth medium or calcification medium. Western blots were carrying out from CSE and CBS double gene silencing utilizing siRNA. D) IL1-β and E) TNF-α western blot was showing. Western blots were normalized to GAPDH. Results were analyzed by One Way ANOVA, Bonferroni’s Multiple Comparison Test and were shown as mean values ± SEM of five independent experiments. *P < 0.01; **P < 0.001; ***P < 0.0001.

As expected, calcification of human valvular interstitial cells also occurred in response to high phosphate. Surprisingly, both IL1-β and TNF-α were induced in human interstitial cells maintained in high phosphate (Fig. 3A and B). Treatment of cells with AP72 significantly inhibited the accumulation of calcium deposits in extracellular matrix, and that was accompanied by the abolishment of the induction of pro-inflammatory cytokines (Fig. 3A-C).

### Reduction of endogenous H_2_S production enhances phosphate-provoked inflammation in valvular interstitial cells

We recently observed that mitigation of endogenous H_2_S production by lowering CSE and CBS expression promotes calcification of valvular interstitial cells [3]. Therefore, to test whether the anti-inflammatory effect of endogenously produced H_2_S also exist we performed experiments employing double silencing of CSE and CBS. We found that using interfering RNAs for CSE/CBS significantly enhanced the expression of both IL1-β and TNF-α in human vascular interstitial cells (Fig. 3A and B) exposed to high phosphate suggesting that H_2_S controls the progress of inflammation under calcifying conditions.

### Suppression of inflammatory processes by H_2_S occurs via inhibiting the activation of NF-κB

**Fig. 4:**
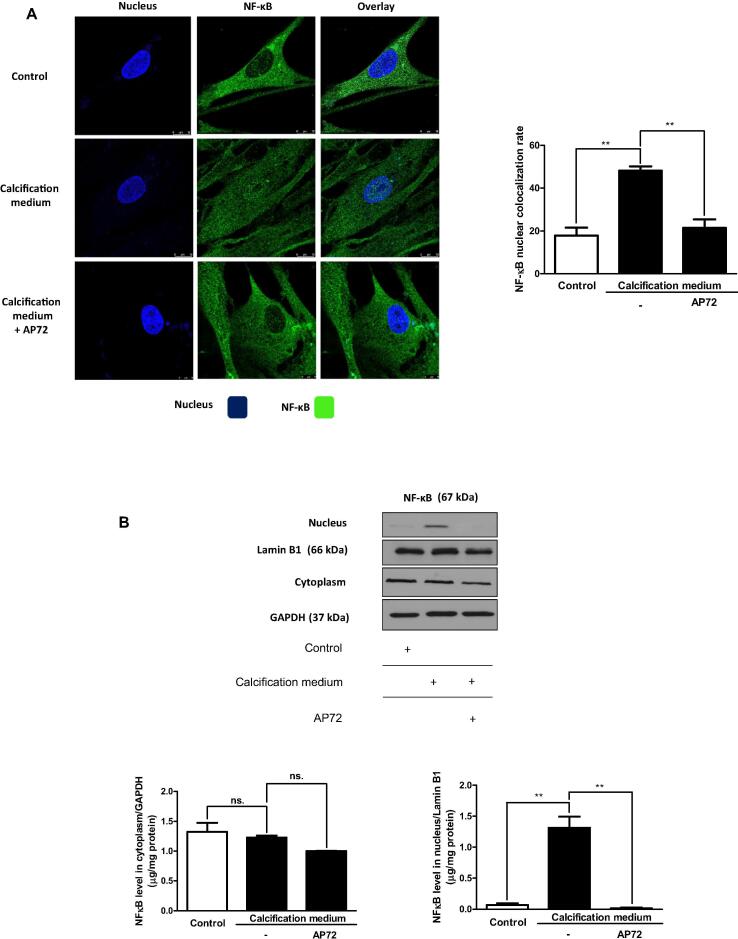
Supplementation of AP72 suppresses nuclear translocation of NF-κB. VIC were grown on coverslips and were exposed to growth medium or calcification medium in the absence or presence with AP72. A) Cells were stained with Hoechst 33,258 for DNA (blue) an anti-NF-κB antibody with Alexa Flour 488 secondary antibody (green). Images were taken with Leica TCS SP8 gated STED-CW nanoscopy. Images were deconvolved using Huygens Professional software. Colocalization rate (right panel) of nucleus/NF-κB was shown in the right panel of Fig. 5A. B) Expression of NF-κB in cytoplasmic and nuclear fraction of VIC was shown. The intensity of bands was normalized to Lamin B1 in case of nuclear extracts and for GAPDH in case of cytoplasm extracts. Results were analyzed by One Way ANOVA, Bonferroni’s Multiple Comparison Test and were shown as mean values ± SEM of five independent experiments. Ns.: not significant; *P < 0.01; **P < 0.001.

It is well established that NF-κB regulates pro-inflammatory signaling pathway and its activation is based on its nuclear translocation followed by pro-inflammatory cytokine expressions such as interleukin 1β (IL-1β) and tumor necrosis factor α (TNF-α) [19]. It has been shown that H_2_S capable of transsulfuration of inhibitor-κB resulting in an inhibition of nuclear translocation of NF-κB [20], [28], [29]. Thus, we examined whether AP72 affects the activation of NF-κB in cells maintained in calcifying condition. Confocal microscopy and immunofluorescence staining indicated that NF-κB was located in the cytoplasm of valvular interstitial cells cultured in growth medium (control medium) (Fig. 4A; upper panels). Phosphate exposure of cells triggered the translocation of NF-κB from the cytoplasm to the nucleus (Fig. 4A; middle panels). As demonstrated in Fig. 4A (lower panel) AP72 prevented the appearance of NF-κB in the nucleus of valvular interstitial cells maintained in calcification medium. To confirm our immunofluorescence finding, cytoplasm and nucleus fractions of valvular interstitial cells were examined for NF-κB employing western blot analysis. We found that NF-κB appeared in the nucleus in response to phosphate exposure (Fig. 4B) while its level was decreased in the cytoplasmic fraction. Importantly, AP72 treatment prevented the translocation of NF-κB into the nucleus of valvular interstitial cells exposed to phosphate (Fig. 4B).

### NF-κB activation triggers Runx2 nuclear translocation and subsequent mineralization of valvular interstitial cells

**Fig. 5:**
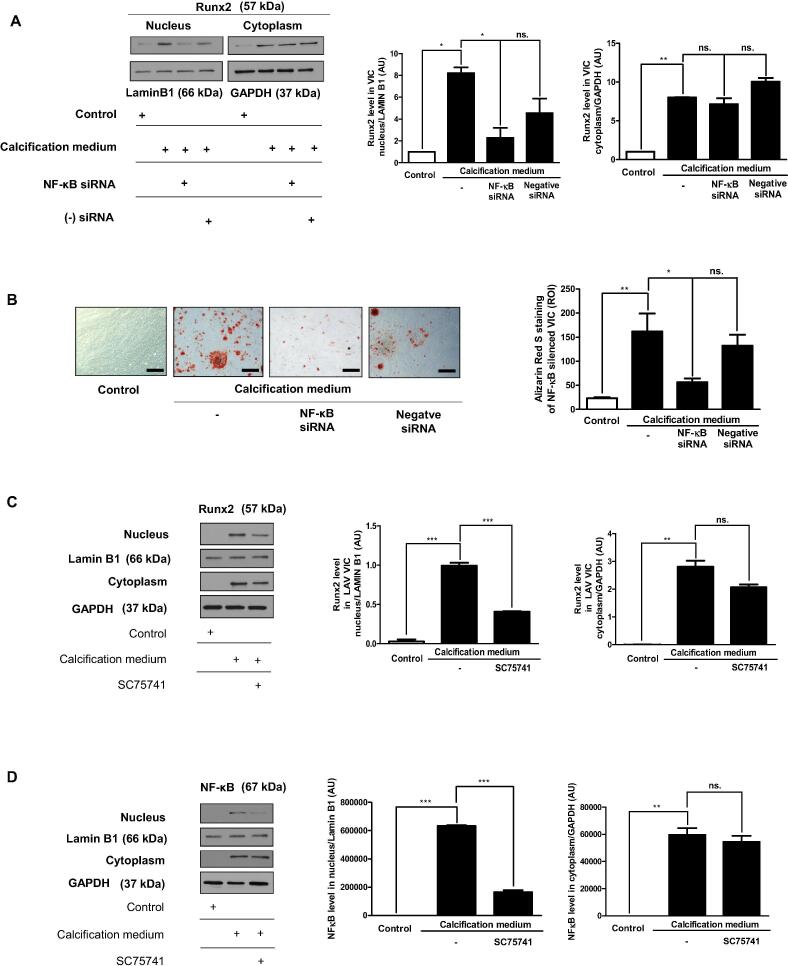
NF-κB gene silencing decreases the nuclear translocation of Runx2. VIC were cultured in growth medium or calcification medium. NF-κB gene silencing using siRNA was performed. A) Runx2 expression in cytoplasmic and nuclear fraction of VIC was shown. The intensity of bands was normalized for Lamin B1 in case of nuclear extracts and for GAPDH in the case of cytoplasm extracts. B) Alizarin Red S staining was shown. Pharmacological inhibition of NF-κB by SC75741 was performed. C) NF-κB and D) Runx2 protein expression in cytoplasmic and nuclear fraction of VIC were shown. The intensity of bands was normalized for Lamin B1 in case of nuclear extracts and for GAPDH in case of cytoplasm extracts. Results were analyzed by One Way ANOVA, Bonferroni’s Multiple Comparison Test and were shown as mean values ± SEM of five independent experiments. Ns.: not significant; *P < 0.01; **P < 0.001; ***P < 0.0001.

Runx2 is a key transcription factor associated with an early osteoblastic differentiation of vascular smooth muscle cells and valvular interstitial cells. We were curious whether there is a link between NF-κB and Runx2 in human valvular interstitial cells in calcific conditions. We cultured human valvular interstitial cells in calcifying media where the NF-κB gene was silenced. As expected nuclear translocation of Runx2 occurred in cells in response to phosphate exposure. To our surprise, silencing NF-κB in human valvular interstitial cells using small interfering RNA (siRNA) markedly decreased nuclear translocation of Runx2 (Fig. 5A). To confirm this observation, we employed a pharmacological inhibitor of NF-κB (SC75741). As shown in Fig. 5C, treatment of cells with the synthetic NF-κB inhibitor SC75741 significantly reduced the nuclear appearance of Runx2 (Fig. 5C). To further collect evidence that inflammatory process in valvular interstitial cells regulates mineralization via NF-κB, we cultured cells in calcifying conditions where the NF-κB gene was silenced and followed the accumulation of calcium in extracellular matrix. Importantly, silencing of NF-κB inhibited deposition of calcium in extracellular matrix of valvular interstitial cells maintained in calcifying media (Fig. 5D).

**Increased expression and nuclear colocalization of NF-κB and Runx2 in valvular interstitial cells occur during aortic valve calcification in apolipoprotein E deficient mice - both are inhibited by H_2_S**

**Fig. 6:**
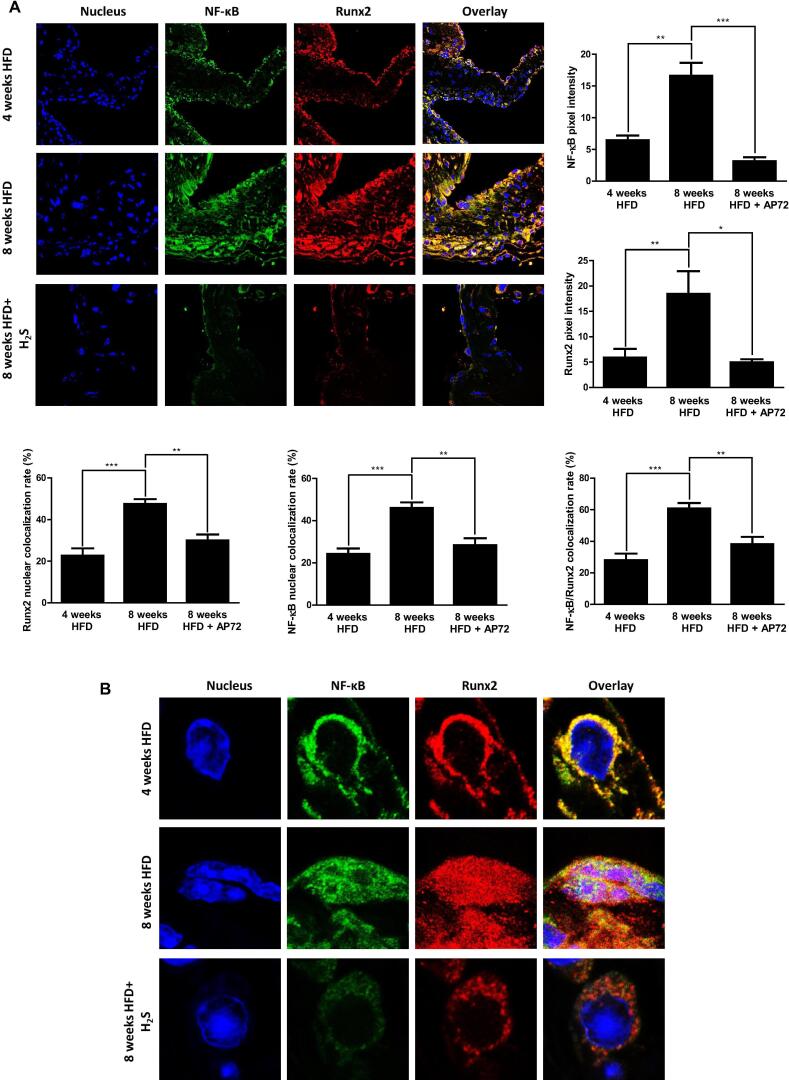
Hydrogen sulfide attenuates NF-κB and Runx2 in heart valves of ApoE^-/-^ mice. ApoE^-/-^ mice fed with high-fat diet. Control mice were injected intraperitoneally with saline for 4 weeks or 8 weeks; H_2_S treated mice were injected intraperitoneally with AP72 for 8 weeks. Heart valves of ApoE^-/-^ mice were stained with Hoechst 33,258 for DNA (blue) an anti-NF-κB antibody with Alexa Flour 488 secondary antibody (green) and an anti-Runx2 antibody with Alexa Flour 647 secondary antibody (red). A) Double immunofluorescence staining of NF-κB and Runx2 was performed. B) High magnification images of mouse heart valve were taken with Leica TCS SP8 gated STED-CW nanoscopy. Images were deconvolved using Huygens Professional software. NF-κB and Runx2 color intensity were shown. Nuclear NF-κB and Runx2 colocalization rate were shown. NF-κB/ Runx2 colocalization rate was shown. Colocalization rates were calculated by ImageJ software. Results were analyzed by One Way ANOVA, Bonferroni’s Multiple Comparison Test and were shown as mean values ± SEM of five independent experiments. *P < 0.01; **P < 0.001; ***P < 0.0001.

In apolipoprotein E deficient mice kept on high-fat diet valvular calcification appears [3] and that is accompanied by valvular inflammation (Fig. 1) as it was earlier revealed in CAVD [30]. Since in our *in vitro* experiments H_2_S inhibited nuclear translocation of both NF-κB and Runx2, and translocation of Runx2 was shown to be dependent on NF-κB, we examined their localization in aortic valves of ApoE^-/-^ mice kept on a high-fat diet with or without administration of AP72. As demonstrated by dual immunohistochemistry the expression of NF-κB and Runx2 was pronounced by 8 weeks in aortic valves of ApoE^-/-^ mice kept on a high-fat diet (Fig. 6A). Treatment of animals with AP72 resulted in significantly less staining for both NF-κB and Runx2 in aortic valve tissue. Confocal microscopy revealed that NF-κB and Runx2 were mainly located in the perinuclear area of cells at 4 weeks, and they were translocated into the nucleus by 8 weeks. Employing STED nanoscopy on valvular samples indicated that NF-κB and Runx2 were colocalized in perinuclear region and the nucleus (Colocalization rate: 60.75% ± 7%). AP72 treatment of ApoE^-/-^ mice kept on a high-fat diet prevented nuclear appearance of NF-κB and Runx2 in valvular interstitial cells (Fig. 6A and B).

## Discussion conclusion

This study is the first that highlights the relationship between NF-κB and Runx2 as a link between inflammation and vascular mineralization, and we report how hydrogen sulfide controls cytokines expression and subsequently abrogates calcification of aortic valve as well as inhibits osteoblastic transformation of valvular interstitial cells. It has been revealed that CAVD is accompanied by calcification and inflammation resulting in heterogeneity of the affected aortic valve tissue. The ratio of the calcified and non-calcified regions provides a guide in distinguishing the state of mineralization [31], [32], [33], [34], [35]. CAVD is an active and well-regulated inflammatory disease, in which inflammation is a critical initiation step involved in valvular disease as shown by the National Heart Lung and Blood Institute [14] and other research groups [9], [14], [16], [35], [36], [37], [38], [39] although, specific molecular mechanisms are not fully understood.

Increasing number of evidences indicates the key role of osteogenic transcription factor Runx2 in cardiovascular calcification including CAVD [3], [14], [30], [40], [41]. For instance, the increase in intracellular phosphate level promotes nuclear translocation of Runx2 resulting in transition of cells towards an osteoblast phenotype [14]. Upregulation of Runx2 was identified to be a maladaptive response during injury to the vasculature, in uremic milieu, and hyperglycemic conditions [42] and was suggested to be both a mediator and a potential therapeutic target in vascular mineralization [43]. In our previous studies [3], [30] and others’ [3], [14], [30], [41], [42] Runx2 was found to be manifested in the nucleus of valvular tissue cells in CAVD indicating the presence of an early signal for transformation of valvular interstitial cells into osteoblast-like cells.

We have shown that endogenous production of H_2_S by CSE and CBS and H_2_S releasing molecules such as AP72 exhibit an inhibition on aortic valve calcification in ApoE^-/-^ mouse and transdifferentiation of human valvular interstitial cells toward osteoblast-like cells [3]. Three separate anti-calcification pathways for H_2_S was observed. i, inhibition of nuclear translocation of Runx2, ii, lowering inorganic phosphate uptake, and iii, increasing pyrophosphate generation. These previous studies and our findings prompted us to examine whether lowering inflammation by H_2_S might contribute to the inhibition of mineralization of aortic valve and such a benefit occur via regulating Runx2 in valvular interstitial cells.

AP72 has an excellent water solubility and very slow generation of H_2_S compared to the fast H_2_S releasing donors such as Na_2_S and NaSH [28], [44], [45]. It is increasingly recognized that slow-releasing H_2_S donors potentially better mimic the effects of the endogenous H_2_S buffer system, because of their slow generation of low sulfide levels [24], [28], [29]. Therefore, we employed AP72 for our experiments. Importantly, exogenous source of H_2_S abrogated inflammation of aortic valves of ApoE^-/-^ mice provoked by a high-fat diet (Fig. 2) as reflected by lowering TNF-α and IL-β. Accordingly, rise of TNF-α and IL-β levels induced by phosphate was abolished by treatment of human valvular interstitial cells with AP72. To investigate whether endogenous H_2_S production has an anti-inflammatory effect, we silenced the expression of CSE and CBS. Since the interaction among CSE/CBS expression was revealed by Nandi and Mishra, demonstrating that CBS deficiency upregulates CSE protein levels [46], we employed double silencing. Reduction of endogenous H_2_S production enhanced phosphate-provoked elevation of TNF-α and IL-β levels in valvular interstitial cells indicating a control of inflammation by the endogenous production of H_2_S.

Expression of pro-inflammatory cytokines, IL-1β and TNF-α are regulated by NF-κB via its nuclear translocation [19]. It has been recently revealed that activation of NF-κB is inhibited by H_2_S [20]. This study prompted us to test whether inflammation and calcification is connected at the level of master transcription factors, NF-κB and Runx2. Corroborate the above findings, AP72 prevented the translocation of NF-κB into the nucleus in valvular interstitial cells exposed to phosphate (Fig. 4). We also observed that activation of Runx2 is inhibited by H_2_S [3]. Therefore, we silenced NF-κB as well as employed in NF-κB inhibitor then followed activation of Runx2 in valvular interstitial cells exposed to phosphate. Importantly, phosphate failed to induce translocation of Runx2 into nuclei in cells lacking NF-κB indicating a link between pro-inflammatory signaling pathway and osteogenic signaling pathway. Using in vivo approaches, we further revealed that the expression of NF-κB and Runx2 were upregulated during aortic valve calcification in ApoE^-/-^ mice and both are prevented by H_2_S (Fig. 6). Strong colocalization was observed between NF-κB and Runx2 in the perinuclear region and the nuclei in valvular interstitial cells during the progression of aortic valve calcification (Fig. 6).

## Conclusion

Our study suggests that regulation of Runx2 by H_2_S (CSE/CBS) occurs via NF-κB resulting in an anti-calcification action and therefore establishing a link between inflammation and mineralization in CAVD.

## Declaration of Competing Interest

The authors declare that they have no known competing financial interests or personal relationships that could have appeared to influence the work reported in this paper.
